# A Specific Urinary Amino Acid Profile Characterizes People with Kidney Stones

**DOI:** 10.1155/2020/8848225

**Published:** 2020-06-30

**Authors:** Aniello Primiano, Silvia Persichilli, Pietro Manuel Ferraro, Riccardo Calvani, Alessandra Biancolillo, Federico Marini, Anna Picca, Emanuele Marzetti, Andrea Urbani, Jacopo Gervasoni

**Affiliations:** ^1^Università Cattolica del Sacro Cuore, Rome, Italy; ^2^Fondazione Policlinico Universitario “Agostino Gemelli” IRCCS, Rome, Italy; ^3^Department of Physical and Chemical Sciences, Università degli Studi dell'Aquila, L'Aquila, Italy; ^4^Department of Chemistry, Sapienza Università di Roma, Rome, Italy

## Abstract

**Background:**

Urolithiasis is the process of stone formation in the urinary tract. Its etiology is only partly known, and efficient therapeutic approaches are currently lacking. Metabolomics is increasingly used in biomarkers discovery for its ability to identify mediators of relevant (patho)physiological processes. Amino acids may be involved in kidney stone formation. The aim of the present study was to investigate the presence of an amino acid signature in stone former urine through a targeted metabolomic approach.

**Methods:**

A panel of 35 amino acids and derivatives was assessed in urines from 15 stone former patients and 12 healthy subjects by UPLC-MS. Partial Least Squares Discriminant Analysis (PLS-DA) was used to define amino acid profiles of cases and controls. *Results and Discussion.* Our approach led to the definition of a specific amino acid fingerprint in people with kidney stones. A urinary amino acid profile of stone formers was characterized by lower levels of *α*-aminobutyric acid, asparagine, ethanolamine, isoleucine, methionine, phenylalanine, serine, tryptophan, and valine. Metabolomic analysis may lend insights into the pathophysiology of urolithiasis and allow tracking this prevalent condition over time.

## 1. Introduction

Urolithiasis is the process of stone formation in different portions of the urinary tract, including the kidneys, bladder, and/or urethra. It represents a worldwide problem associated with high healthcare costs due to surgical interventions for its resolution and subsequent medical care [1–4]. Management of urolithiasis is complex and suffers three main issues: (a) its high prevalence, (b) the high probability of recurrence [5], and (c) the lack of effective interventions, either dietary or pharmacological [6]. The etiology of the disease is only partly known. Urolithiasis is caused by the formation of crystals in the urinary tract, when the urine becomes supersaturated, due to a reduced urine volume or excessive excretion of solutes. The aggregation of crystals and their growth lead to the formation of stones that can present different compositions. Urinary and kidney stones are most commonly composed by calcium oxalate mono- and dihydrate, calcium phosphate, ammonium urate, magnesium ammonium phosphate, calcium hydrogen phosphate dihydrate, uric acid, and its salts and cysteine [7–9]. The crystallization process may be modulated by promoters or inhibitors of crystallization or by the presence of a matrix of crystalline material present in the renal papilla (Randall's plaques), found in most patients with calcium urinary stones [10]. Another important indicator of the disease is the onset of relapses, which defines a specific “metabolic activity” in patients predisposed to the formation of multiple kidney stones. Although knowledge on relapse risk factors and pathogenesis has increased [11, 12], the early identification of patients at higher risk of relapse is not yet possible, although relative supersaturation estimates have been shown to predict the risk of recurrence [13].

Metabolomics is the study of small molecules present in a cell, tissue, or organism that result from the metabolic processes occurring in both physiologic and pathologic conditions [14]. Metabolomics has therefore become a cornerstone approach in biomarker discovery and for the development of personalized medicine strategies [15].

Both targeted and untargeted metabolomic approaches have been used in nephrology research to identify novel markers of kidney disease and its complications [16–18]. Recently, a NMR-based metabolomic study found that four metabolic pathways, including glyoxylate and dicarboxylate metabolism; glycine, serine, and threonine metabolism; phenylalanine metabolism; and citrate cycle (TCA cycle), were closely associated with kidney stone [19].

The aim of the present investigation was to determine the amino acid profile of a group of patients with urolithiasis and healthy controls through a targeted UPLC-MS method coupled with multivariate chemometric analysis. This approach may provide novel insights into the role played by protein/amino acid metabolism in kidney stone formation and candidate biomarkers for the early diagnosis of urolithiasis.

## 2. Material and Methods

### 2.1. Study Participants

This pilot study was conceived as a cross-sectional, case-control investigation. Briefly, after obtaining written informed consent, twenty-four-hour urine samples were collected from a small group of patients hospitalized for lithiasis (stone formers, SF) in the Division of Nephrology of IRCCS Policlinico Gemelli Foundation and were analyzed in the Division of Laboratory Diagnostic Area. All patients were recurrent stone formers with no active pharmacological treatment at the time of evaluation. Secondary causes of renal lithiasis were excluded. For patients who had recently undergone an endourological procedure or ureteral stent removal, the metabolic evaluation was performed after at least 3 weeks from the procedure. Healthy subjects without history of kidney stones or major urological problems were taken as controls (CNT). After collection, all urinary aliquots were immediately stored at −80°C until analysis.

### 2.2. Chemicals and Reagents

Amino acid standards were purchased from Sigma (Saint Louis, MS, USA). Isotopically labeled amino acid standards were from Cambridge Isotope Laboratories (Andover, MA, USA). AccQ-Tag Ultra eluent concentrates and an AccQ-Tag Ultraderivatization kit were purchased from Waters Corporation (Milford, MA, USA). Acetonitrile was from (Merck KGaA, Germany). Deionized water was from (Merck KGaA, Germany).

### 2.3. Amino Acid Determination

For the metabolomic analysis, the sample purification and derivatization were carried using the AccQ-Tag kit (Waters Corporation, USA) according to manufacturer instructions. Briefly, 50 *μ*L of sample was mixed with 100 *μ*L of 10% (*w*/*v*) sulfosalicylic acid containing an internal standard mix (50 *μ*M) and centrifuged at 1000 × *g* for 15 min. Ten *μ*L of the supernatant was transferred into a vial containing 70 *μ*L of borate buffer to which 20 *μ*L of AccQ-Tag reagents (Waters Corporation, Milford, MA) was subsequently added. Samples were then vortexed for 10 s and heated at 55°C for 10 min. The chromatographic separation was performed by ACQUITY H-Class (Waters Corporation) using an ACQUITY CORTECS C18 column (Waters Corporation) eluted at a flow rate of 500 *μ*L/min with a linear gradient (9 min) from 99 to 1 water 0.1% formic acid in acetonitrile 0.1% formic acid. MS was an ACQUITY QDa single quadrupole equipped with an electrospray source operating in positive mode (Waters Corporation). The analytical process was monitored using amino acid controls (level 1 and level 2) manufactured by the MCA laboratory of the Queen Beatrix Hospital (The Netherlands). Urinary amino acid concentrations were determined by comparison with values obtained from a standard curve for each amino acid (0,5-2,5-125-250-500 *μ*mol/L for all amino acids only for cystine 1-5-50-250-500-1000 *μ*mol/L). Through this method, it is possible to assess simultaneously 35 amino acids (alanine, *α*-aminobutyric acid, aminoadipic acid, anserine, arginine, asparagine, aspartic acid, *β*-alanine, *β*-aminobutyric acid, carnosine, citrulline, cystine, ethanolamine, *γ*-aminobutyric acid, glycine, glutamic acid, histidine, isoleucine, 4-hydroxyproline, leucine, lysine, methionine, 1-methylhistidine, 3-methylhistidine, ornithine, phenylalanine, phosphoethanolamine, proline, sarcosine, serine, taurine, threonine, tryptophan, tyrosine, and valine) in several biological matrices such as urine, plasma, and saliva. For data analysis (calibration curves and amino acid quantitation), the instrument software TargetLynx was used.

## 3. Statistical Data Analysis

### 3.1. Univariate Analysis

Comparisons between SFs and CNTs for normally distributed continuous variables were performed by *t*-test statistics. Mann-Whitney *U* test was applied to quantify differences for nonnormally distributed continuous data.

Descriptive analyses were performed using the GraphPrism 5.03 software (GraphPad Software, Inc., San Diego, CA), with a statistical significance set at *p* < 0.05. All values obtained were expressed as mean ± standard error of mean (SEM). Mann-Whitney *U* test was performed to compare the difference in the means between the stone formers and controls. A *p* value < 0.05 was considered as statistically significant.

### 3.2. Multivariate Analysis

The strategy pursued to detect biomarkers for kidney stone formation starts from the calculation of a classification model. Partial Least Squares Discriminant Analysis was used to distinguish people with kidney stones (i.e., cases) from the other enrollees (i.e., controls). Then, two variable selection approaches, variable importance in projection (VIP) and rank product (RP), were used to detect the analytes contributing the most to the characterization of the amino acidic pattern of case patients. Finally, the statistical significance of the classification model has been investigated through an inspection of the number of misclassification (NMC), the area under the receiver operating characteristic curve (AUROC), and the discriminant Q2 (DQ2).

### 3.3. Partial Least Squares Discriminant Analysis (PLS-DA)

Discriminant classifiers allow assigning samples to specific categories or classes. In this work, Partial Least Squares Discriminant Analysis (PLS-DA) [20, 21] was used to identify people with kidney stones and discriminating them from the controls. This classification method is advisable in this framework, and it has been used in similar contexts [22–24] because, among the other advantages, it is suitable for handling correlated variables as the ones investigated in the present study. Namely, PLS-DA exploits PLS regression to find a set of latent variables (called scores) maximizing the correlation between the predictors (i.e., the original features collected on the investigated individuals) and a response matrix encoding the class belongings [25].

The accuracy of the classification model was internally validated, applying a double cross-validation (DCV) procedure [26].

### 3.4. Biomarker Selection

VIP [27] and RP [28] were used to detect which biomarker contributes the most to the characterization of people with nephrolithiasis and their distinction from control cases. In the present work, these indices have been calculated as described by Calvani et al. [29, 30].

### 3.5. Evaluation of the Statistical Significance through a Permutation Tests

The statistical significance of the PLS-DA results was investigated through the inspection of the number of misclassification (NMC), the area under the receiver operating characteristic curve (AUROC), and the discriminant Q2 (DQ2) under the null hypothesis calculated through a 1000-repetition permutation test [31]. The significance threshold used is *p* value < 0.05.

## 4. Results and Discussion

Fifteen SFs and twelve CNTs were enrolled in the study. The main demographic and anthropometric characteristics of the study population according to the classification groups are presented in Table 1. Stone formers and controls were different by age and body composition/BMI; for this reason, we had extensively investigated whether this could lead to an important bias for the results or not, collecting enough evidences which allowed to rule out such possibility (see Figures S1, S2, and S3).

**Figure 1:**
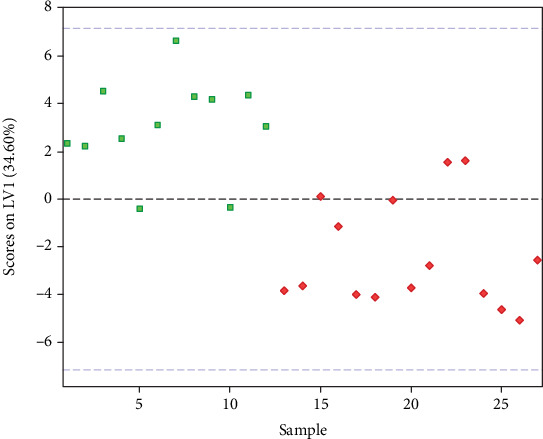
Score plot showing the separation of participants (stone formers in green; healthy controls in red) on the space determined by the LVs according to PLS-DA model. LV: latent variable; PLS-DA: Partial Least Squared-Discriminant Analysis.

A double cross-validated PLS-DA model was run in order to evaluate the presence of a distinct amino acid profile in people with renal lithiasis compared with control patients. The best PLS-DA model was built using two LVs. A good classification performance was obtained by the selected PLS-DA model. Indeed, we were able to correctly classify 84.2 ± 3.6% of the study participants (82.2 ± 4.7% of cases and 86.7 ± 4.7% of controls) in the internal DCV loop used for model selection and 75.7 ± 3.2% (76.0 ± 5.1% of cases and 75.3 ± 4.1% of controls) in the outer DCV loop, which results from repeated cycles of external validation steps. The differences in the amino acid profiles of cases and controls as well as the evident classification performance of the PLS-DA model are apparent when inspecting the projection of the study participants' scores over the space spanned by the LVs (Figure 1).

VIP and RP were used for detecting which amino acids contribute the most to the solution of the classification problem. The first nine biomarkers detected by the two approaches are the same, indicating these analytes definitely characterize the nephrolithiasis condition. In particular, stone formers showed lower levels of *α*-aminobutyric acid, asparagine, ethanolamine, isoleucine, methionine, phenylalanine, serine, tryptophan, and valine, than healthy controls (Table 2).

Serum concentrations of nondiscriminant analytes according to the PLS-DA model are reported in Table S1.

**Figure 2:**
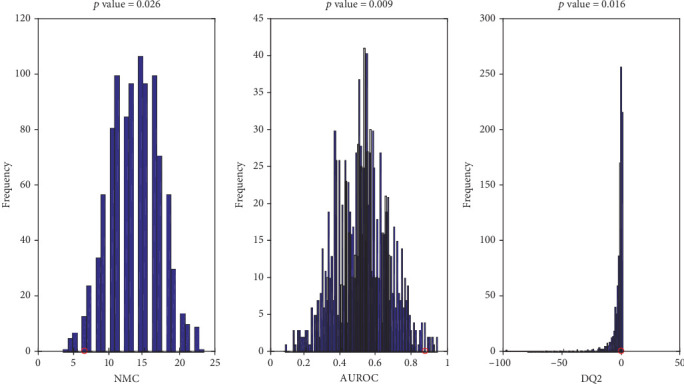
Values obtained on the real dataset (red circles) fall outside of the corresponding null hypothesis distribution (blue histograms), corresponding to a *p* < 0.05.

In order to test the statistical significance of the PLS-DA model, NMC, AUROC, and DQ2 under the null hypothesis were calculated through the 1000-repetition permutation test (Figure 2). The outcome of this further investigation is that the results obtained by the PLS-DA model are statistically significant. In fact, inspecting Figure 2, it is straightforward that, regardless the figure of merit investigated, the results provided by the classification model on (unpermuted) real data (red circles) fall on the edge of the null hypothesis distribution, leading to *p* values of 0.026, 0.009, and 0.016 for NMC, AUROC, and DQ2, respectively.

Stone formers are more likely to have more significant urinary and metabolic abnormalities compared with nonstone formers [32]. At the moment, there are no reliable biomarkers to predict risk of recurrent stones. 24-hour urine and plasma routine analysis as part of the clinical evaluation does not always accurately predict stone recurrence, although their execution should be performed as recommended by clinical guidelines. In this scenario, metabolomic analyses of urine using innovative targeted approaches may help develop new diagnostic and therapeutic algorithms to better predict urinary stone recurrence.

Our preliminary results suggest that several metabolic pathways are perturbed in nephrolithiasis. In particular, the presence of low urinary levels of ethanolamine, serine, and tryptophan seems to corroborate previous findings on people with kidney stones [19]. Alterations in amino acid levels and other metabolic abnormalities may be relevant to kidney stone formation. It is acknowledged that perturbations of oxalate, calcium, citrate, and cystine metabolism are associated to urolithiasis [33, 34]. In this context, altered urinary levels in stone formers may be suggestive of altered glycine metabolism that could lead to increased oxalate production and stone formation [35]. Our findings are in agreement with literature data [35–39], in which a decrease in the concentration of amino acids, including alanine, tryptophan, and threonine, was suggested to exert a possible inhibitory activity of crystalline aggregation [38, 39]. Moreover, renal oxidative vulnerability due to changes in mitochondrial-glutathione and energy homeostasis was described in a rat model of calcium oxalate urolithiasis [40]. Interestingly, a role for *α*-aminobutyric acid has been hypothesized in the regulation of glutathione biosynthesis following oxidative stress stimulation [41]. Indeed, parallel to the activation of glutathione synthesis, the production of its analogue ophthalmic acid from *α*-aminobutyric acid is initiated [42]. The reduced urinary levels of *α*-aminobutyric acid found in people with kidney stones may thus be the resultant of increased oxidative stress and/or a perturbed glutathione biosynthetic process [41].

Although reporting novel findings, the present study has some limitations.

First, the population that was investigated was relatively small, and numerous experimental variables were measured. However, the analytical approach that was used, i.e., PLS-DA plus a double cross-validation, is particularly suited to handle such an experimental setting and provide stringent and interpretable estimation of statistical significance.

Some factors might affect circulating amino acid concentration. For instance, gut microbiota acts as a metabolic modulator in nephrolithiasis condition and the decrease of certain metabolites can be associated with disruption of the intestinal microbiota due to exposure to antibiotic therapies. Different levels of circulating amino acid can also be possible due to diet and lifestyle [43–45]. Neither the amount of physical activity nor nutritional patterns were quantified in the present study.

However, as recently showed, differences in circulating amino acid levels seem to be less marked than those resulting from nutritional interviews [43]. The cross-sectional nature of our study does not allow inference on cause-effect relationships.

## 5. Conclusion

In this pilot study, we showed that a urinary amino acid fingerprint exists in people with kidney stones. Such an experimental approach may be used to improve our understanding of urolithiasis and, after a thorough validation in independent cohorts, be used in the clinical evaluation of this condition in addition to metabolic routine analysis and nutritional assessment.

## Figures and Tables

**Table 1:** Main characteristics of people involved in the study, according to the presence of urolithiasis.

	CNT (*n* = 12)	SF (*n* = 15)
Male (*n* (%))	4 (47)	9 (60)
Age	32 (21-56)	57 (30-70)
BMI (kg/m^2^)	20.9 (17.6-26.0)	25.1 (21.3-36.3)

Data are expressed as median (min-max value) for continuous variables and count (%) for categorical ones. Abbreviations: BMI: body mass index; CNT: controls; SF: stone formers.

**Table 2:** The urine average levels of discriminant amino acids in patients and the control group.

AA	CNT		SF		*p* value
	Average	±SD	Average	±SD	
*α*-Aminobutyric acid	14.0	5.8	6.1	2.5	<0.0001
Asparagine	214.5	73.2	71.4	44.0	<0.0001
Ethanolamine	365.4	69.9	215.3	87.6	0.0003
Isoleucine	9.9	3.7	4.2	3.8	0.0029
Methionine	8.2	2.8	2.7	2.7	0.0003
Phenylalanine	49.5	13.2	24.7	15.4	<0.0001
Serine	354.1	109.0	105.6	93.8	<0.0001
Tryptophan	61.2	14.3	33.5	21.5	0.0023
Valine	34.4	12.0	15.2	11.4	0.0025

## Data Availability

All data are available in the article.

## Supplementary Materials

Supplementary Materials Figure S1: univariate correlation between the candidate markers and age. Figure S2: univariate correlation between the candidate markers and BMI. Figure S3: cross-validated results of multivariate calibration of age or BMI vs. candidate markers of stone formation. Table S1: the urine average levels of amino acids in patients and the control group.

## References

[1] Jr Scales C. D., Smith A. C., Hanley J. M., Saigal C. S., Urologic Diseases in America Project (2012). Prevalence of Kidney Stones in the United States. *European Urology*.

[2] Croppi E., GEA Firenze Study Group, Ferraro P. M., Taddei L., Gambaro G. (2012). Prevalence of renal stones in an Italian urban population: a general practice-based study. *Urological Research*.

[3] Strohmaier W. L. (2019). Economics of stone disease/treatment. *Arab Journal of Urology*.

[4] Ferraro P. M., Vittori M., Macis G. (2018). Changes in renal papillary density after hydration therapy in calcium stone formers. *BMC Urology*.

[5] Ferraro P. M., Curhan G. C., D’Addessi A., Gambaro G. (2017). Risk of recurrence of idiopathic calcium kidney stones: analysis of data from the literature. *Journal of Nephrology*.

[6] Ferraro P. M., Taylor E. N., Gambaro G., Curhan G. C. (2017). Dietary and Lifestyle Risk Factors Associated with Incident Kidney Stones in Men and Women. *The Journal of Urology*.

[7] Primiano A., Persichilli S., Gambaro G. (2014). FT-IR Analysis of Urinary Stones: A Helpful Tool for Clinician Comparison with the Chemical Spot Test. *Disease Markers*.

[8] Primiano A., Persichilli S., Ferraro P. M. (2019). A combination of infrared spectroscopy and morphological analysis allows successfully identifying rare crystals and atypical urinary stones. *Ann Ist Super Sanità*.

[9] Gervasoni J., Primiano A., Ferraro P. M., Urbani A., Gambaro G., Persichilli S. (2018). Improvement of Urinary Stones Analysis Combining Morphological Analysis and Infrared Spectroscopy. *Journal of Chemistry*.

[10] García-Perdomo H. A., Solarte P. B., Espana P. P. (2016). Pathophysiology associated with forming urinary stones. *Urología Colombiana*.

[11] Bargagli M., Primiano G., Primiano A. (2019). Recurrent kidney stones in a family with a mitochondrial disorder due to the m.3243A>G mutation. *Urolithiasis*.

[12] De Paolis E., Minucci A., De Bonis M. (2018). A rapid screening of a recurrent CYP24A1 pathogenic variant opens the way to molecular testing for Idiopathic Infantile Hypercalcemia (IIH). *Clinica Chimica Acta*.

[13] Ferraro P. M., Ticinesi A., Meschi T. (2018). Short-term changes in urinary relative supersaturation predict recurrence of kidney stones: a tool to guide preventive measures in urolithiasis. *Journal of Urology*.

[14] Cacciatore S., Loda M. (2015). Innovation in metabolomics to improve personalized healthcare. *Annals of the New York Academy of Sciences*.

[15] Krzyszczyk P., Acevedo A., Davidoff E. J. (2019). The growing role of precision and personalized medicine for cancer treatment. *Technology*.

[16] Rhee E. P. (2018). A Systems-Level View of Renal Metabolomics. *Seminars in Nephrology*.

[17] Abbiss H., Maker G., Trengove R. (2019). Metabolomics Approaches for the Diagnosis and Understanding of Kidney Diseases. *Metabolites*.

[18] Dubin R. F., Rhee E. P. (2020). Proteomics and Metabolomics in Kidney Disease, including Insights into Etiology, Treatment, and Prevention. *Clinical Journal of the American Society of Nephrology*.

[19] Duan X., Zhang T., Ou L., Kong Z., Wu W., Zeng G. (2020). 1H NMR-based metabolomic study of metabolic profiling for the urine of kidney stone patients. *Urolithiasis*.

[20] Sjöström M., Wold S., Söderström B., Gelsema E. S., Kanal L. N. (1986). PLS discriminant plots. *Pattern Recognition in Practice*.

[21] Ståhle L., Wold S. (1987). Partial least squares analysis with cross‐validation for the two‐class problem: A Monte Carlo study. *Journal of Chemometrics*.

[22] Calvani R., Picca A., Marini F. (2018). The "BIOmarkers associated with Sarcopenia and PHysical frailty in EldeRly pErsons" (BIOSPHERE) study: Rationale, design and methods. *European Journal of Internal Medicine*.

[23] Calvani R., Picca A., Marini F. (2018). A Distinct Pattern of Circulating Amino Acids Characterizes Older Persons with Physical Frailty and Sarcopenia: Results from the BIOSPHERE Study. *Nutrients*.

[24] Marzetti E., Picca A., Marini F. (2019). Inflammatory signatures in older persons with physical frailty and sarcopenia: The frailty "cytokinome" at its core. *Experimental Gerontology*.

[25] Barker M., Rayens W. (2003). Partial least squares for discrimination. *Journal of Chemometrics*.

[26] Filzmoser P., Liebmann B., Varmuza K. (2009). Repeated double cross validation. *Journal of Chemometrics*.

[27] Wold S., Johansson E., Cocchi M., Kubinyi H. (1993). PLS - partial least-squares projections to latent structures. *3D QSAR in drug design: Theory, methods and applications*.

[28] Smit S., van Breemen M. J., Hoefsloot H. C. J., Smilde A. K., Aerts J. M. F. G., de Koster C. G. (2007). Assessing the statistical validity of proteomics based biomarkers. *Analytica Chimica Acta*.

[29] Calvani R., Brasili E., Praticò G. (2014). Fecal and urinary NMR-based metabolomics unveil an aging signature in mice. *Experimental Gerontology*.

[30] Calvani R., Marini F., Cesari M. (2015). Biomarkers for physical frailty and sarcopenia: state of the science and future developments. *Journal of Cachexia, Sarcopenia and Muscle*.

[31] Szymańska E., Saccenti E., Smilde A. K., Westerhuis J. A. (2012). Double-check: validation of diagnostic statistics for PLS-DA models in metabolomics studies. *Metabolomics*.

[32] D'Alessandro C., Ferraro P. M., Cianchi C., Barsotti M., Gambaro G., Cupisti A. (2019). Which Diet for Calcium Stone Patients: A Real-World Approach to Preventive Care. *Nutrients*.

[33] Khan S. R., Pearle M. S., Robertson W. G. (2016). Kidney stones. *Nature Reviews Disease Primers*.

[34] Jiang J., Knight J., Easter L. H., Neiberg R., Holmes R. P., Assimos D. G. (2011). Impact of dietary calcium and oxalate, and Oxalobacter formigenes colonization on urinary oxalate excretion. *The Journal of Urology*.

[35] Golovanova O. A., Korolkov V. V., Punin Y. O., Vysotskiy A. S. (2013). Effect of Amino Acids on the Crystallization Kinetics of Calcium Oxalate Monohydrate. *Chemistry for Sustainable Development*.

[36] Atanassova S. S., Panchev P., Ivanova M. (2010). Plasma levels and urinary excretion of amino acids by subjects with renal calculi. *Amino Acids*.

[37] Atanassova S. S. (2014). Influence of the lysine on the calcium oxalate renal calculi. *International Urology and Nephrology*.

[38] Taranets Y. V., Bezkrovnaya O. N., Pritula I. M., Mateychenko P. V. (2017). L-Threonine Amino Acid as a Promoter of the Growth of Pathogenic Calcium Oxalate Monohydrate Crystals. *Journal of Nanomaterials & Molecular Nanotechnology*.

[39] Gao S., Yang R., Peng Z. (2016). Metabolomics analysis for hydroxy-L-proline-induced calcium oxalate nephrolithiasis in rats based on ultra-high performance liquid chromatography quadrupole time-of-flight mass spectrometry. *Science Reports*.

[40] Meimaridou E., Lobos E., Hothersall J. S. (2006). Renal oxidative vulnerability due to changes in mitochondrial-glutathione and energy homeostasis in a rat model of calcium oxalate urolithiasis. *American Journal of Physiology-Renal Physiology*.

[41] Irino Y., Toh R., Nagao M. (2016). 2-Aminobutyric acid modulates glutathione homeostasis in the myocardium. *Science Reports*.

[42] Soga T., Baran R., Suematsu M. (2006). Differential metabolomics reveals ophthalmic acid as an oxidative stress biomarker indicating hepatic glutathione consumption. *The Journal of Biological Chemistry*.

[43] Ticinesi A., Nouvenne A., Chiussi G., Castaldo G., Guerra A., Meschi T. (2020). Calcium Oxalate Nephrolithiasis and Gut Microbiota: Not Just a Gut-Kidney Axis. A Nutritional Perspective. *Nutrients*.

[44] Schmidt J. A., Rinaldi S., Scalbert A. (2016). Plasma concentrations and intakes of amino acids in male meat-eaters, fish-eaters, vegetarians and vegans: a cross-sectional analysis in the EPIC-Oxford cohort. *European Journal of Clinical Nutrition*.

[45] Fukai K., Harada S., Iida M. (2016). Metabolic Profiling of Total Physical Activity and Sedentary Behavior in Community-Dwelling Men. *PLoS One*.

