# Supplementary material for: A Specific Urinary Amino Acid Profile Characterizes People with Kidney Stones
Source: Dis Markers. 2020 Jun 30;2020:8848225. doi: 10.1155/2020/8848225 (PMC7345965; doi:10.1155/2020/8848225)
Supplement: Supplementary Materials — Figure S1: univariate correlation between the candidate markers and age. Figure S2: univariate correlation between the candidate markers and BMI. Figure S3: cross-validated results of multivariate calibration of age or BMI vs. candidate markers of stone formation. Table S1: the urine average levels of amino acids in patients and the control group. [file 8848225.f1.docx]

**Supplementary Materials**

Figure S1 – Univariate correlation between the candidate markers and age.

Figure S2 – Univariate correlation between the candidate markers and BMI.

As it can be observed from both Figures S1 and S2, age and BMI are rather weakly correlated to the concentration of those aminoacids which have been identified as potential metabolic biomarkers for the stone forming conditions.

Figure S3 – Cross-validated results of multivariate calibration of age or BMI vs candidate markers of stone formation.

It is evident from the Figure how the aminoacids identified as relevant for predicting the stone forming condition correlate very weakly, also when considered altogether in a multivariate model, with the potential covariates age and BMI. These observations allow to rule out with a rather strong support the possibility that differences in age and BMI between cases and control may induce any bias in the classification results.

Table S1: The urine average levels of amino acids in patients and control group

|  |  |  |  |  |  |
| --- | --- | --- | --- | --- | --- |
| **AA** | **CNT** | | **SF** | |  |
|  | **average** | **± SD** | **average** | **± SD** | **p value** |
| Alanine | 325.7 | 105.4 | 182.8 | 96.9 | 0.0019 |
| Aminoadipic acid | 40.6 | 19.5 | 30.7 | 14.8 | 0.2941 |
| Anserine | 43.6 | 65.5 | 25.3 | 35.9 | 0.1184 |
| Arginine | 14.1 | 5.0 | 8.6 | 5.4 | 0.0112 |
| Aspartic acid | 3.0 | 1.6 | 4.2 | 4.2 | 1000.0 |
| β -alanine | 28.1 | 34.9 | 17.2 | 15.0 | 0.6084 |
| β -aminobutyric acid | 60.3 | 18.6 | 49.7 | 35.1 | 0.1128 |
| Carnosine | 7.9 | 6.4 | 19.8 | 22.2 | 0.0263 |
| Citrulline | 2.1 | 0.8 | 1.6 | 1.7 | 0.0599 |
| Cystine | 76.1 | 40.9 | 35.1 | 18.7 | 0.0037 |
| γ -aminobutyric acid | 1.7 | 1.3 | 1.5 | 1.4 | 0.3785 |
| Glycine | 1478.4 | 721.9 | 695.6 | 643.6 | 0.0043 |
| Glutamic acid | 29.2 | 34.3 | 20.3 | 20.8 | 0.1303 |
| Histidine | 805.1 | 277.1 | 397.6 | 307.0 | 0.0032 |
| 4-hydroxyproline | 2.9 | 5.6 | 4.7 | 4.9 | 0.0066 |
| Leucine | 25.8 | 9.7 | 10.7 | 11.1 | 0.0046 |
| Lysine | 221.4 | 179.5 | 80.8 | 89.5 | 0.0120 |
| 1 methyl histidine | 858.0 | 819.2 | 657.8 | 469.8 | 0.9029 |
| 3 methyl histidine | 290.2 | 103.9 | 299.9 | 106.4 | 0.6429 |
| Ornithine | 10.9 | 5.5 | 7.7 | 3.5 | 0.1184 |
| Phosphoethanolamine | 17.2 | 8.8 | 16.6 | 8.2 | 0.8836 |
| Proline | 5.0 | 2.4 | 4.9 | 5.1 | 0.2941 |
| Sarcosine | 2.1 | 1.5 | 1.4 | 1.0 | 0.2124 |
| Taurine | 692.2 | 194.3 | 680.3 | 853.9 | 0.0338 |
| Threonine | 132.5 | 36.7 | 310.0 | 846.4 | 0.0094 |
| Tyrosine | 82.6 | 28.2 | 44.1 | 28.4 | 0.0068 |
|  |  |  |  |  |  |
